# Exploring Association Between Social Media Addiction, Fear of Missing Out, and Self-Presentation Online Among University Students: A Cross-Sectional Study

**DOI:** 10.3389/fpsyt.2022.896762

**Published:** 2022-05-13

**Authors:** Xinhong Zhu, Zhenfang Xiong

**Affiliations:** School of Nursing, Hubei University of Chinese Medicine, Wuhan, China

**Keywords:** social media addiction, self-presentation, fear of missing out, university students, privacy setting

## Abstract

**Background:**

Social media addiction (SMA) is known to be associated with excess use of social media. However, few studies have focused on the links of self-presentation on social media, fear of missing out (FoMO) and SMA. The present study investigated the relationships of self-presentation, FoMO and SMA among university students.

**Methods:**

Online survey was conducted with 2,744 respondents, who completed online survey including social media use, FoMO and SMA. Self-presentation on social media and privacy information protection were assessed via researcher-designed questionnaires. Self-presentation on social media was composed of basic information shown on social media and expression willingness. Privacy information protection contained information viewed by others and privacy settings in social media platforms.

**Results:**

The most common information posted on social media were gender, hobby, age, personal photos, videos, and birthday. The most common social platforms with privacy setting were QQ zone (62.2%), WeChat (60.1%), and QQ (40.3%). FoMO (*OR* = 2.852, *P* = 0.000), information viewed by others (*OR* = 4.261, *P* = 0.000), managing a personal homepage (OR = 1.339, *P* = 0.002), accept a stranger's “friend request” (*OR* = 1.251, *P* = 0.028) and undergraduate students and above (*OR* = 1.439, *P* = 0.001) predicted expression willingness. FoMO (*OR* = 5.278, *P* = 0.000), information viewed by others (*OR* = 9.673, *P* = 0.000), privacy setting in QQ (*OR* = 0.817, *P* = 0.002) and in Tik Tok (*OR* = 0.536, *P* = 0.019) and female (*OR* = 0.588, *P* = 0.004) significantly influenced basic information shown on social media. Furthermore, FoMO (*OR* = 4.165, *P* = 0.000), expression willingness (*OR* = 1.645, *P* = 0.000), and information viewed by others (*OR* = 1.406, *P* = 0.000) positively affected the level of SMA. Risk of SMA increased as time spent on social media per day. However, basic information shown on social media did not significantly influence SMA.

**Conclusion:**

In general, students with higher level of FoMO and expression willingness are more likely to experience SMA. These results highlight individual behaviors on social media should be considered as essential elements for assessing problematic engaging to social media.

## Introduction

Social media has emerged as an integral part of individuals' daily lives in the cyber age. As of June 2020, there is about 970 million netizens in China (1). Additionally, the greatest number of Chinese netizens are students, accounting for 23.7%, and the proportion of Internet users among young people aged 20-29 has been estimated to be 19.9% (1). Regarding social applications in China, 85.0% of social network users are active on the Moments in WeChat, with 41.6% using Qzone and 40.4% using sina Weibo (1). Social media enables people to make new friends and maintain relationship without geographical or time constraints, access to information and find funny or entertaining contents (2, 3), but excessive usage and the achieved pleasure leading to addiction (4, 5). Against this circumstance, a critical issue has been seriously raised concerning the potential implications of social media use on Chinese university students' mental health and wellbeing (6). The fact that drawing attention is occurrence of mental disorders and Social Media Addiction (SMA) (4–8).

SMA is defined as paying excessive attention to social media activities often to the neglect of all other activities, and uncontrollable use to the extent that it interferes with other important areas of life including psychological health, interpersonal relationships, emotional consequences, academic performance, and occupation to the detriment of the individual (9–12). A number of studies related SMA are guided by motivation-related theories, such as the uses and gratifications theory and the self-determination theory (13). Social gratifications (e.g., maintaining relationships, interacting with others, and receiving social support) and sociopsychological needs (e.g., need to belong and need for relatedness) are major types of motivations predicting addictive social media use (14, 15). Myriad studies have investigated the problematic social media use typically accompanied by a reduction in a size of the individuals' social circle, as well as an increase in loneliness, depression, and fear of missing out (FoMO) (10, 16–18). FoMO demonstrates moderate-to-large relations with levels of excessive social media use (19, 20). Given social media as an inseparable part of university students' daily life, concerns have been raised about its excessive usage globally (21–23). Students with SMA are overly concerned about social media driven by a strong motivation to log on to or use social media and devote much time and effort to social media (24). However, the relationship of FoMO and SMA is still further explored.

In addition, individual's may use social media exclusively for information acquisition (25) and self-presentation (26), both of which may contribute to SMA. The major motivation of social media use is self-presentation for managing a personal homepage (27, 28). Self-presentation is defined as the goal-directed activity of controlling information to influence the impressions formed by an audience about the self (29). Social media activities that accomplish self-presentational goals include posting photographs, profile information, impression management, and self-expression, which are important aspects of relational development in maintaining relationship (27–30). Using social media for social interaction seems to have positive effects on an individual's online self-presentation (31, 32). Whereas, the excessive and inappropriate use of social media for entertainment purpose might decrease willingness to express themselves (32, 33). The inconsistency of relationship between social media use and self-presentation seems to due to the different purposes of social media usage. Although previous researches have focused on the important role of purposes of social media use on self-presentation, less attention is paid to the association between negative consequences of inappropriate social media use and self-presentation. The aim of the present study is to explore the relationships of FoMO, SMA and self-presentation on social media and determine the factors affecting SMA in university students.

## Methods

### Sample and Data Collection

Using convenience sampling 3,015 participants from four universities and four vocational and technical in Wuhan comprising junior college students, undergraduate students, and graduate students. The criteria for involvement in the research were being voluntary to participate in the study and cooperate with the study. Data were collected using structured-questionnaires in February, 2021. Participants filled out questionnaires online via Questionnaire Star. Data from 3,015 were used for analysis; 271 respondents were excluded due to missing data.

### Instruments

#### Chinese Social Media Addiction Scale

The scale was developed by (34), original used with college students in China. The tool contains 28 items evaluated on a five-point likert scale ranging from one strongly disagree to five strongly agree. Cronbach's α was 0.971 in this study. High score indicates a high level of SMA. The participants were divided into two groups using the mean SMA score for statistical analysis: a high score group (≥84, *n* = 1,376) and a low score group (<84, *n* = 1,368).

#### Fear of Missing Out Scale (FoMOs)

the Chinese version of FoMOs, which was adapted by Li et al. following standardized international guidelines, was implemented to measure FoMO among university students in China (35, 36). Responses for each item are rated by using a five-point likert scale ranging from one (strongly disagree) to five (strongly agree). The Cronbach's α value of FoMOs in the present study was 0.924. High scores indicate high level of FoMO. The participants were divided into two groups using the mean FoMO score for statistical analysis: a high score group (≥36, *n* = 975) and a low score group (<36, *n* = 1,769).

#### Self-Presentation on Social Media

The tool contains two parts as followed: (1) Expression willingness contains five items (share or repost, comment, reply, like, original article). Responses for each item are rated by using a five-point likert scale ranging from one (strongly disagree) to five (strongly agree). The Cronbach's α values of expression willingness in the present study was 0.913. High scores indicate a high level of expression willingness on social media. The participants were divided into two groups using the mean expression willingness score for statistical analysis: a high score group (≥15, *n* = 1,000) and a low score group (<15, *n* = 1,744). (2) Basic information shown on social media contains 15 items (real-time position, social relations, consumption information, personal photos, and videos, location, newsletter, education, emotional state, marriage, height, and weight, birthday, hobby, gender, age, and name). Responses for each item are rated by using a five-point Likert scale ranging from one (strongly disagree) to five (strongly agree). The Cronbach's α value of self-information posted on social media in the present study was 0.927. High score indicates a high level of basic information shown on social media. The participants were divided into two groups using the mean basic information shown on social media score for statistical analysis: a high score group (≥45, *n* = 2,480) and a low score group (<45, *n* = 264).

#### Privacy Information Protection

The tool contains two questions as followed: (1) Item one is privacy setting in social platforms (WeChat, Weibo, QQ, QQ zone, Tik Tok, Little Red Book, Douban, Post bar, Zhi Hu, others). Responses for each item are rated by using “Yes or No”; (2) Item two is information viewed by others. Can your social friends get your following information online? (Consumption information, hobby, location, education, occupation, age, and gender). Responses for each item are rated by using a five-point likert scale ranging from one (strongly disagree) to five (strongly agree). The Cronbach's α value of information viewed by others in the present study was 0.915. High scores indicate high level of information online viewed by others. The participants were divided into two groups using the mean information viewed by others score for statistical analysis: a high score group (≥21, *n* = 1,454) and a low score group (<21, *n* = 1,290).

#### Social Media Use

Social media use is ascertained with the following items: (1) Social media platforms; (2) Purposes of using social media; (3) Category of information online you prefer; (4) Purposes of updating social feed; (5) Number of social media accounts; (6) People who interact most frequently on social media; (7) The motivations for interacting with others most frequently; (8) Accept a stranger's “friend request”; (9) Time spent on social media (h); (10) Browsing social media before going to bed; (11) Do you spend more time on social networking than real world?

#### Demographic Questionnaire

A demographic information sheet is used to acquire basic information, such as gender, age, residence, education, single-child, and parental marital status.

### Ethical Consideration

The study was conducted according to the guidelines of the Declaration of Helsink, and approved by the Institutional Ethical Review Committee of Hubei University of Chinese Medicine (2018-ICE-023). Prior to the collection of data, the purposes and procedures of this study were explained to the respondents. Participants were informed that they would withdraw from the study at any time. Data were collected only from those who voluntarily agreed and provided written consent to participate in the study.

### Data Analysis

All analyses were performed using SPSS 20.0, and the significance level of statistical tests were set up *P* < 0.05. Descriptive analysis of participants' demographic characteristics and social media use were described using number (*n*), percentage (%), and mean ± standard deviation (*SD*). The score of SMA was normal distribution. The Spearman correlation was used to determine the relationship of SMA, FoMO, and self-presentation on social media. Logistic regression was fitted to identify significant factors (*P* < 0.05) associated with SMA, social media use, privacy information protection, FoMO, expression willingness, and basic information shown on social media. The detailed binary regression steps were as followed: (1) In model one, FoMO, and privacy information protection was entered to detect influencing factors on expression willingness or basic information shown on social media. (2) In model two, FoMO, privacy information protection, and social media use were entered to detect significant factors on expression willingness or basic information shown on social media. (3) In model three, FoMO, privacy information protection, social media use, and demographic information were entered. The strength of association was explained in terms of odds ratio (*OR*) and a 95% confidence interval (CI). Meanwhile, binary regression was fitted to access affecting factors on SMA, after considering the effects of FoMO, privacy information protection, expression willingness, or basic information shown on social media, social media use, and demographic information. The models' goodness of fit was check using omnibus tests of model coefficients for overall fitness of the model and Hosmer and Lemeshow test for fitness of the data to the model.

## Results

### Sample Characteristics

Sample characteristics were shown in Table 1. The mean age of participants was 20.08 (*SD* = 2.37) with a range of 18-30 years old. Of the 2,744, 69.2% were female, 69.4% were single child and 56.3% lived in rural. Most participants were undergraduate students and above (58.2%). Ten point three percentage of students were from single parent or stepparent families.

**Table 1:** General characteristics of the participants (*n* = 2,744).

**Variables**	***n***	**%**
**Gender**		
Female	1,899	69.2
Male	845	30.8
**Residence**		
City	1,199	43.7
Rural	1,545	56.3
**Single-child**		
Yes	1,905	69.4
No	839	30.6
**Education**		
Junior college students	1,147	41.8
Undergraduate students and above	1,597	58.2
**Marital status of parents**		
Married	2,410	87.8
Single parent or stepparent	284	10.3
Others	50	1.8

### Social Media Use and Privacy Information Protection

**Figure 1:**
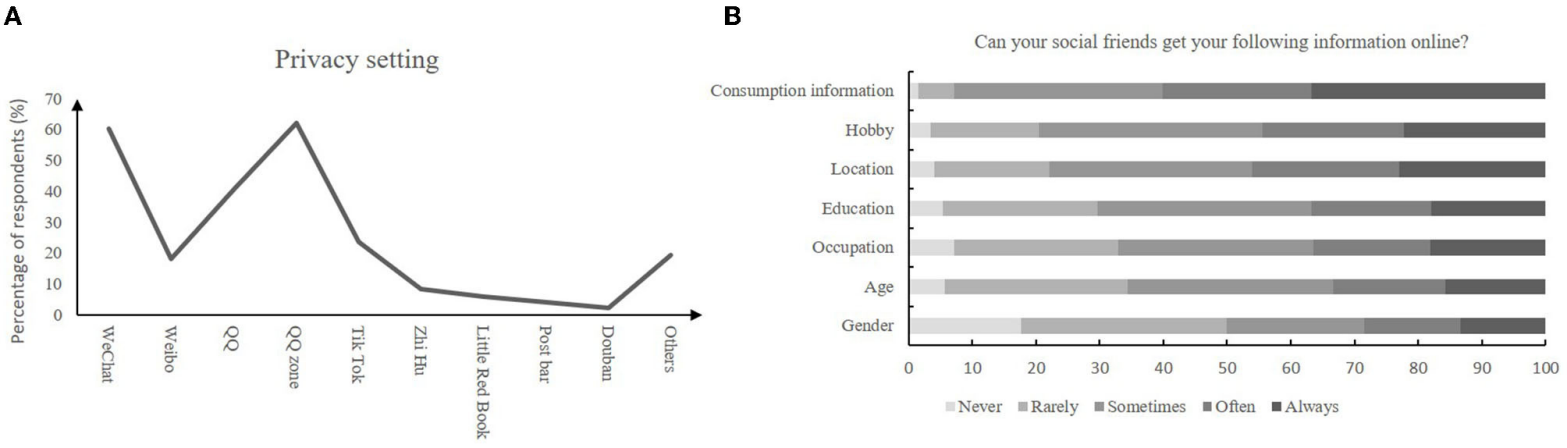
Privacy information protection. **(A)** Privacy setting; **(B)** Information viewed by others.

As shown in Supplementary Table S1, 41.6% respondents admitted that they spend more time on social networking than real world. Regarding the question “can your social friends get your following information online?”, nearly two or three students reported their social friends could know their following information from their social media platforms: gender, age, occupation, location, and education level (Figure 1A). Moreover, nearly two or three students reported that they had privacy settings on WeChat and Qzone (Figure 1B). Sixty point six percentage students accepted a stranger's “friend request”.

### Self-Presentation on Social Media

**Figure 2:**
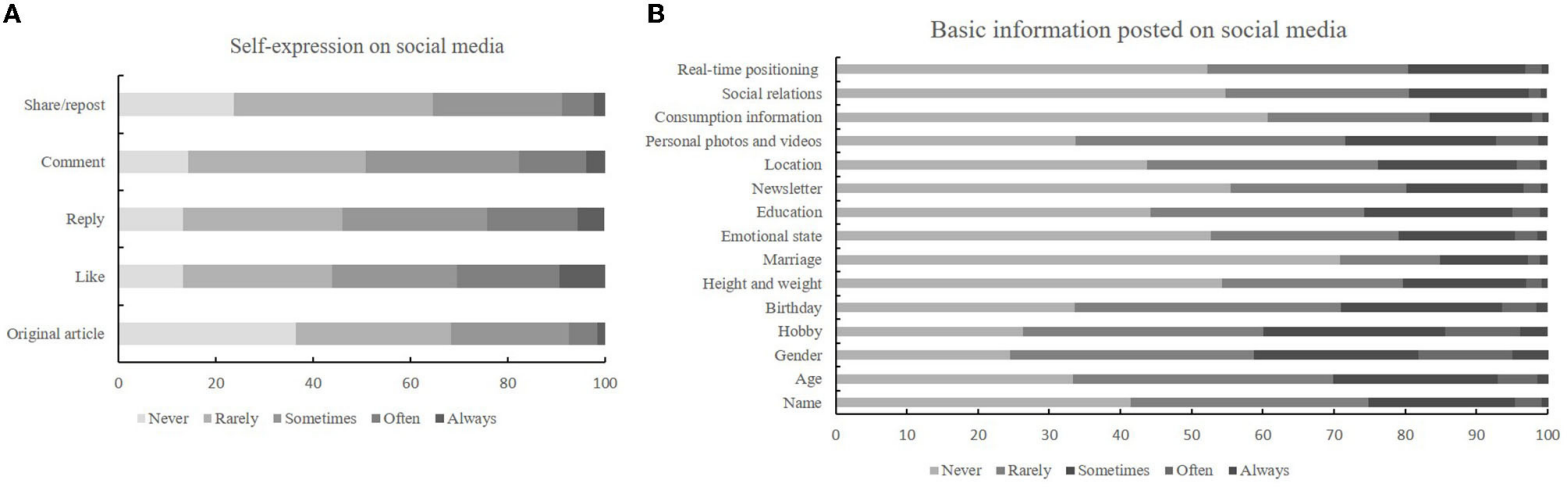
Individual self-presentation on social media. **(A)** Expression willingness; **(B)** Basic information posted on social media.

As depicted in Figure 2A, regarding the question “Expression willingness,” almost one or four students admitted that they “sometimes” gave a like, shared/reposted, or replied, and 24.3% participants reported they “sometimes” published original article. Furthermore, a total of 16.3% students reported that they “sometimes” showed real-time positioning on social media, while 37.9% of them indicated they “often” posted photos, or videos (Figure 2B). Accept a stranger's “friend request” (*OR* = 1.251), FoMO (*OR* = 4.261), privacy setting [QQ *(OR* = 0.775) and Weibo (*OR* = 0.430)] and information viewed by others (*OR* = 2.852) affected the level of expression willingness (Table 2). Among male university, accept a stranger's “friend request” and education level did not influence expression willingness (Supplementary Table S2). Furthermore, FoMO (*OR* = 5.278), information viewed by others (*OR* = 9.673), and privacy setting [QQ (*OR* = 0.817), and Tik Tok (*OR* = 0.546)] affected level of basic information posted on social media (Table 3). Among male university, parents who interact most frequently on social media negatively affect basic information shown on social media (Supplementary Table S3).

**Table 2:** Factors associated with expression willingness among university students.

**Variable**	**Model 1**	**Model 2**	**Model 3**
	**OR, 95% CI**	**OR, 95% CI**	**OR, 95% CI**
FoMO	2.993 (2.502–3.581)^**^	2.810 (2.325–3.423)^**^	2.852 (2.348–3.464)^**^
Information viewed by others	4.485 (3.722–5.404)^**^	4.355 (3.578–5.318)^**^	4.261 (3.492–5.200)^**^
**Privacy setting**			
Weibo	0.392 (0.180–0.595)^*^	0.428 (0.253–0.692)^*^	0.430 (0.248–0.698)^*^
QQ	0.745 (0.612–0.906)^*^	0.776 (0.628–0.959)^*^	0.775 (0.626–0.958)^*^
**Social media platform**			
**Weibo**		1.467 (1.175–1.832)^*^	1.424 (1.136–1.785)^*^
**Purposes of using social media**			
To stay up–to–date with news and current events		1.304 (1.043–1.629)^*^	1.296 (1.035–1.624)^*^
To share photos or videos with others		1.295 (1.028–1.631)^*^	1.301 (1.032–1.640)^*^
**Category of information online you prefer**			
Friends' updates		1.310 (1.042–1.647)^*^	1.313 (1.044–1.653)^*^
**Purposes of updating social feed**			
Managing a personal homepage		1.338 (1.078–1.660)^*^	1.339 (1.078–1.663)^*^
No purposes		0.775 (0.621–0.968)^*^	0.768 (0.614–0.960)^*^
**People who interact most frequently on social media**			
Relatives		0.776 (0.604–0.997)^*^	0.798 (0.620–0.998)^*^
Netizens		1.495 (1.174–1.904)^*^	1.426 (1.105–1.840)^*^
Accept a stranger's “friend request”		1.249 (1.026–1.521)^*^	1.251 (1.027–1.524)^*^
**Time spent on social media (h)**			
0–2		Ref	Ref
2–4		1.413 (1.064–1.877)^*^	1.420 (1.068–1.888)^*^
4–6		1.379 (1.014–1.875)^*^	1.420 (1.042–1.937)^*^
6–8		1.567 (1.059–2.318)^*^	1.599 (1.077–2.372)^*^
8–		1.278 (0.823–1.984)	1.363 (0.874–2.126)
Undergraduate students and above			1.439 (1.171–1.769)^*^
Hosmer and Lemeshow Test	0.163	0.488	0.314

* *P < 0.05*;

** *P < 0.001*.

*Model 2: adjusted social media use; Model 3: additionally, adjusted demographic information*.

**Table 3:** Factors associated with basic information shown on social media among university students.

**Variable**	**Model 1**	**Model 2**	**Model 3**
	**OR, 95% CI**	**OR, 95% CI**	**OR, 5% CI**
FoMO	5.899 (4.268–8.153)^**^	5.667 (3.979–8.072)^**^	5.278 (4.013–8.175)^**^
Information viewed by others	8.508 (5.289–13.686)^**^	9.379 (5.718–15.385)^**^	9.673 (5.872–15.933)^**^
**Privacy setting**			
WeChat	1.418 (1.005–2.000)^*^	1.426 (1.070–2.097)^*^	1.508 (0.918–2.234)
QQ	0.716 (0.519–0.988)^*^	0.805 (0.561–0.991)^*^	0.817 (0.568–0.998)^*^
Tik Tok		0.536 (0.435–0.897)^*^	0.546 (0.455–0.907)^*^
**Social media platform**			
QQ		0.483 (0.275–0.846)^*^	0.453 (0.268–0.766)^*^
Tik Tok		0.677 (0.476–0.969)^*^	0.647 (0.457–0.915)^*^
**Purposes of using social media**			
Playing game		1.429 (1.013–2.017)^*^	1.373 (0.970–1.944)
Because a lot of my friends are on them		1.653 (1.096–2.492)^*^	1.619 (1.076–2.456)^*^
**Category of information online you prefer**			
News		0.694 (0.482–0.998)^*^	0.695 (0.481–1.003)
Products		0.555 (0.341–0.903)^*^	0.584 (0.359–0.948)^*^
**Purposes of updating social feed**			
Follow others		2.837 (1.727–4.662)^**^	2.769 (1.677–4.573)^**^
**The motivations for interacting with others most frequently**			
Share information		1.544 (1.349–1.848)^*^	1.536 (1.344–1.837)^*^
**Time spent on social media (h)**			
0–2		Ref	Ref
2–4		1.229 (0.759–1.998)	1.250 (0.770–2.029)
4–6		1.233 (0.734–2.072)	1.307 (0.775–2.204)
6–8		1.938 (1.044–3.529)^*^	2.093 (1.122–3.905)^*^
8–		1.774 (1.289–3.540)^*^	1.938 (1.368–3.880)^*^
Female			0.588 (0.408–0.849)^*^
Hosmer and Lemeshow Test	0.106	0.341	0.710

* *P < 0.05*;

** *P < 0.001*.

*Model 2: adjusted social media use*;

*Model 3: additionally, adjusted demographic information*.

### SMA Status

The mean score of SMA was 77.76 (*SD* = 19.65). The results revealed a positive significant correlation between SMA and FoMO (*r* = 0.544, *P* = 0.00), between SMA and expression willingness (*r* = 0.397, *P* = 0.000). As depicted in Table 4, FoMO (*OR* = 4.165), expression willingness (*OR* = 1.645), information viewed by others (*OR* = 1.406) positively affected the level of SMA scores. However, basic information posted on social media did not influence SMA scores. Furthermore, risk of SMA increased as time spent on social media daily and longer than real world increased. Among female university students, using social media for shopping negatively influenced SMA (Supplementary Table S4).

**Table 4:** Factors associated with SMA among university students.

**Variable**	**Model 1**	**Model 2**	**Model 3**
	**OR, 95% CI**	**OR, 95% CI**	**OR, 95% CI**
FoMO	4.482 (3.708–5.417)^**^	4.241 (3.287–5.063)^**^	4.165 (3.404–5.097)^**^
Expression willingness	1.909 (1.574–2.316)^**^	1.749 (1.341–2.028)^**^	1.645 (1.336–2.026)^**^
Basic information shown on social media	1.142 (0.812–1.606)	1.156 (0.821–1.628)	1.217 (0.866–1.776)
Information viewed by others	1.482 (1.240–1.772)^*^	1.445 (1.234–1.764)^**^	1.406 (1.166–1.705)^**^
**Purposes of using social media**			
Playing games		1.301 (1.067–1.581)^*^	1.278 (1.048–1.559)^*^
**Number of social media accounts**			
0–2		Ref	Ref
3–4		1.042 (0.845–1.286)	1.032 (0.836–1.275)
5–6		1.261 (0.945–1.684)	1.234 (0.922–1.650)
7–8		0.889 (0.553–1.430)	0.881 (0.547–1.418)
9–		1.628 (1.046–2.536)^*^	1.605 (1.027–2.509)^*^
**Browsing social media before going to bed**			
Strong disagree		Ref	Ref
Disagree		1.403 (0.656–2.590)	1.352 (0.676–2.702)
Not agree		1.703 (0.837–3.069)	1.687 (0.876–3.251)
Agree		1.907 (1.433–3.500)^*^	1.886 (1.469–3.672)^*^
Strong agree		2.464 (1.160–4.421)^*^	2.348 (1.196–4.610)^*^
**Time spent on social media (h)**			
0–2		Ref	Ref
2–4		1.541 (1.112–1.868)^*^	1.457 (1.122–1.890)^*^
4–6		1.579 (1.112–1.967)^*^	1.519 (1.138~2.026)^*^
6–8		1.744 (1.133–2.387)^*^	1.685 (1.157–2.454)^*^
8–		2.118 (1.695–2.516)^*^	2.067 (1.023–2.401)^*^
**Do you spend more time on social networking than real world?**			
Less		Ref	Ref
The same		1.469 (1.144–1.861)^*^	1.463 (1.147–1.867)^*^
Slightly		1.751 (1.349–2.273)^**^	1.749 (1.346–2.271)^*^
Much		2.223 (1.607–3.065)^**^	2.219 (1.604–3.070)^*^
Hosmer and Lemeshow Test	0.243	0.313	0.235

* *P < 0.05*;

** *P < 0.001*.

*Model 2: adjusted social media use*;

*Model 3: additionally, adjusted demographic information*.

## Discussion

The present study aimed to investigate the association of self-presentation on social media, FoMO, and SMA in two respects: (a) assess the effect of social media use, FoMO, privacy information protection, on self-presentation on social media; (b) exam the effect of FoMO, privacy information protection, and self-presentation on social media on SMA and pay attention to other influencing factors. The over pattern result confirms the effect of self-presentation and FoMO on SMA. Social media usage is partially motivated by a need for a positive self-presentation (37). Consistence with previous studies, using social media for social interaction might render individuals to increase willingness to express their personal and intimate information, such as updating self-descriptive profiles, sharing content within the circle of friends, and giving thumbs-ups (31, 32). Furthermore, self-presentation on social media showed a positive association with SMA in this study. In addition, information posted on social media, which could be viewed by others, also acted as predictor of SMA. That is, people experience higher levels of positive social feedback, which in turn is reflected in an increase in SMA, a process that is interpretable in light of craving for positive social interactions (28–39). Accordingly, self-presentation on social media could significantly predict SMA in university students.

Expression willingness is an interpersonal skill that selects appropriate actions and verbal expressions to communicate one's feelings (40). In this study, sharing photos or videos with others, and managing a personal homepage predicted expression willingness on social media, which is consist with previous study (27, 28). However, no purpose for updating social feed maybe reduce individual's expression willingness. QQ and Weibo were the most popular social media platforms among university students in this study. When QQ and Weibo were set privacy protection, the desire for expression and basic information posted on social media were reduced. Privacy control is found to have a negative impact on personal information posted on social media (41). There is evidence suggesting when communicating anonymously, self-presentation occurs online (42). That's why when university students accepted a stranger's “friend request” and communicated with netizens, they were glad to express on social media in this study. In this study, because of my friends using social media or following others exerted significant influence on personal information posted on social media. Evidence shows that interactivity, privacy control, and trust influence social media users' self-disclosure behavior (43). Trusting friends' choosing plays an important role on personal information shown on social media. Meanwhile, sharing information acting as self-disclosure goal positively influenced the level of basic information posted on social media, which is line with previous study (44).

In keeping with previous studies (33, 45), our study supported the relationship between FoMO and SMA. Next, we found FoMO acted as predictor of SMA and self-presentation on social media. Evidence showed that adolescents with a high level of FoMO would choose to disclose themselves on cyberspace, especially social media to enhance social relationships with other contacts (46). Meanwhile, a high focus on self-presentation on social media is associated with more mental health problems, such as anxiety and depression (47). Therefore, subjects with high levels of FoMO tended to display themselves on social media in order to keep in touch with others in this study. SMA motivated by social interaction and communication check their social media accounts and communicate with social media friends in an automatic and impulsive fashion (48, 49). Thus, FoMO, who are desire to stay continually connected with what others are doing, enhances individuals' state of self-presentation on social media and facilitates SMA.

In this view, the results of this study indicated that SMA is related to the number of social media accounts and time spent on social media, which are in line with previous literatures (50, 51). Furthermore, time spent on social media and browsing social media before going to bed acted as predictors of SMA. With regard to social media use, it has been highlighted that individuals with excessive social media use are more prone to engage in SMA (52).

Gender differences, as well as some similarities, are apparent in factors influencing basic information shown on social media. Female and male differ to some extent in the types of content posted online (53). In this study, the service information that male university students preferred online positively affect basic information shown on social media. Female are more like to display friendship, whereas, male are more likely to orient toward technology, sports, and humor in the information they post to their profile (53). Meanwhile, female are greater concerns about privacy and identity disclosure on social media than male (54). That's why privacy setting acted as opposite role on basic information shown on social media.

## Limitations

The study has limitations. First, the use of a non-random sample of social media users from China, limiting generalizability of results to other countries. Second, some of the studied variables (e.g., FoMOs and SMA) were reported using self-administered questionnaires. Therefore, our findings may suffer from the negative effects of recall bias and social desirability. Finally, a cross-sectional design was used, which cannot provide evidence of the causal relationships among studied variables.

## Conclusions

To conclude, the study provides novel insights into the link of FoMO, self-presentation on social media, and SMA. Particularly, it highlights that addictive social media use is related to habits of social media usage (e.g., browsing social media before going to bed, number of social media accounts and self-presentation on social media), time spent on social media, FoMO and spending more time on social networking than real world. Further research is needed to employ longitudinal designs, which might further verify the underlying mechanisms linking self-presentation and SMA.

## Data Availability Statement

The raw data supporting the conclusions of this article will be made available by the authors, without undue reservation.

## Ethics Statement

The studies involving human participants were reviewed and approved by the Institutional Ethics Review Committee of Hubei University of Chinese Medicine. The patients/participants provided their written informed consent to participate in this study.

## Author Contributions

ZX and XZ: conceptualization, validation, investigation, and project administration. XZ: methodology, formal analysis, data curation, writing—original draft preparation, visualization, and funding acquisition. ZX: writing—review editing and supervision. All authors contributed to the article and approved the submitted version.

## Funding

This research was funded by the National Natural Science Foundation of China (No. 82003448).

## Conflict of Interest

The authors declare that the research was conducted in the absence of any commercial or financial relationships that could be construed as a potential conflict of interest.

## Publisher's Note

All claims expressed in this article are solely those of the authors and do not necessarily represent those of their affiliated organizations, or those of the publisher, the editors and the reviewers. Any product that may be evaluated in this article, or claim that may be made by its manufacturer, is not guaranteed or endorsed by the publisher.
