# Supplementary material for: Exploring Association Between Social Media Addiction, Fear of Missing Out, and Self-Presentation Online Among University Students: A Cross-Sectional Study
Source: Front Psychiatry. 2022 May 13;13:896762. doi: 10.3389/fpsyt.2022.896762 (PMC9136033; doi:10.3389/fpsyt.2022.896762)
Supplement: Supplementary file 1 [file Table_1.docx]

Table S1 Social use of the participants (n = 2744)

| Variable | n (%) | Variable | n (%) |
| --- | --- | --- | --- |
| Social media platform |  | Browsing social media before going to bed |  |
| WeChat | 2569 (93.6) | Strongly disagree | 74 (2.7) |
| Weibo | 1138 (41.5) | Disagree | 282 (10.3) |
| QQ | 2564 (93.4) | Not agree | 927 (33.8) |
| QQ zone | 1054 (38.4) | Agree | 813 (29.6) |
| Tik Tok | 1394 (50.8) | Strongly agree | 648 (23.6) |
| Little Red Book | 785 (28.6) | People who interact most frequently on social media |  |
| Purposes of using social media |  | Teachers | 357 (13.0) |
| To stay in touch with what my friends are doing | 2316 (84.4) | Parents | 1047 (38.2) |
| To research/find products to buy | 1754 (63.9) | Relatives | 760 (27.7) |
| To find funny or entertaining contents | 1787 (65.1) | Friends | 2459 (89.6) |
| Learning | 1777 (64.8) | Schoolmates | 2141 (78.0) |
| To stay up-to-date with news and current events | 1707 (62.2) | Netizens | 582 (21.2) |
| Playing game | 1265 (46.1) | Strangers | 175 (6.4) |
| To share photos or videos with others | 952 (34.7) | Others | 173 (6.3) |
| To initiate a topic | 525 (19.1) | The motivations for interacting with others most frequently | |
| Because a lot of my friends are on them | 479 (17.5) | Obtain information | 1691 (61.6) |
| Others | 314 (11.4) | Share information | 2208 (80.5) |
| Number of social media accounts |  | Consult questions | 1259 (45.9) |
| 0~2 | 833 (30.4) | Maintain relationship | 1672 (60.9) |
| 3~4 | 1214 (44.2) | Do not participate in | 88 (3.2) |
| 5~6 | 436 (15.9) | Others | 422 (15.4) |
| 7~8 | 116 (4.2) | Purposes of updating social feed |  |
| 9~ | 145 (5.3) | Let others know your recent situation | 1130 (41.2) |
| Time spent on social media (h) |  | Managing a personal homepage | 32.9 (90.4) |
| 0~2 | 516 (18.8) | Follow others | 226 (8.2) |
| 3~4 | 1020 (37.2) | No purposes | 857 (31.2) |
| 5~6 | 743 (27.1) | Others | 452 (16.5) |
| 7~8 | 267 (9.7) | Accept a stranger’s “friend request” | 1664 (60.6) |
| 9~ | 198 (7.2) | Category of information online you prefer |  |
| Do you spend more time on social networking than real world? |  | Friends’ updates (Yes) | 1887 (68.8) |
| Less | 636 (23.2) | News (Yes) | 1612 (58.7) |
| The same | 969 (35.3) | Entertainments (Yes) | 1710 (62.3) |
| Slightly | 745 (27.2) | Services (Yes) | 853 (31.1) |
| Much | 394 (14.4) | Knowledge (Yes) | 1949 (71.0) |
|  |  | Health information (Yes) | 1060 (38.6) |
|  |  | Sports (Yes) | 429 (15.6) |
|  |  | Goods (Yes) | 476 (17.3) |
