# Supplementary material for: Exploring Association Between Social Media Addiction, Fear of Missing Out, and Self-Presentation Online Among University Students: A Cross-Sectional Study
Source: Front Psychiatry. 2022 May 13;13:896762. doi: 10.3389/fpsyt.2022.896762 (PMC9136033; doi:10.3389/fpsyt.2022.896762)
Supplement: Supplementary file 2 [file Table_2.docx]

Table S2 Comparison of variables by gender across expression willingness

|  | Male (n = 845) | Female (n = 1899) |
| --- | --- | --- |
|  | OR (95%CI) | OR (95%CI) |
| FoMO | 5.400 (3.589~8.125) ** | 2.319 (1.813~2.937) ** |
| Information viewed by others | 9.580 (6.121~14.993) ** | 3.493 (2.752~4.434) ** |
| Privacy setting |  |  |
| QQ | 1.090 (0.692~1.715) | 0.738 (0.571~0.955) * |
| Social media platform |  |  |
| Weibo | 0.936 (0.545~1.609) | 1.619 (1.246~2.104) ** |
| Purposes of using social media |  |  |
| To stay up-to-date with news and current events | 1.465 (1.196~2.395) * | 1.134 (1.064~1.488) * |
| To share photos or videos with others | 0.960 (0.574~1.608) | 1.462 (1.113~1.920) * |
| Category of information online you prefer |  |  |
| Friends’ updates | 1.546 (0.940~2.543) | 1.280 (1.072~1.684) * |
| Purposes of updating social feed |  |  |
| Managing a personal homepage | 1.504 (0.908~2.490) | 1.434 (1.115~1.845) * |
| No purposes | 0.752 (0.457~1.237) | 0.749 (0.576~0.974) * |
| People who interact most frequently on social media |  |  |
| Relatives | 0.922 (0.513~1.656) | 0.717 (0.534~0.946) * |
| Netizens | 1.621 (1.189~2.658) * | 1.523 (1.106~2.097) * |
| Accept a stranger’ “friend request” | 1.073 (0.702~1.640) | 1.341 (1.181~1.570) * |
| Time spent on social media (h) |  |  |
| 0~2 | Ref |  |
| 2~4 | 1.151 (0.691~1.915) | 1.453 (1.180~2.155) * |
| 4~6 | 1.779 (1.083~3.220) * | 1.598 (1.101~2.321) * |
| 6~8 | 1.241 (0.495~3.114) | 1.807 (1.122~2.909) * |
| 8~ | 0.994 (0.365~2.706) | 1.618 (0.946~2.769) |
| Undergraduate students and above | 1.109 (0.702~1.753) | 1.527 (1.197~1.946) * |
| Hosmer and Lemeshow Test | 0.410 | 0.789 |

*: *P*<0.05; **: *P*<0.001
