# Supplementary material for: Exploring Association Between Social Media Addiction, Fear of Missing Out, and Self-Presentation Online Among University Students: A Cross-Sectional Study
Source: Front Psychiatry. 2022 May 13;13:896762. doi: 10.3389/fpsyt.2022.896762 (PMC9136033; doi:10.3389/fpsyt.2022.896762)
Supplement: Supplementary file 3 [file Table_3.docx]

Table S3 Comparison of variables by gender across basic information shown on social media

|  | Male (n = 845) | Female (n = 1899) |
| --- | --- | --- |
|  | OR (95%CI) | OR (95%CI) |
| FoMO | 6.191 (3.120~12.282) ** | 6.706 (4.227~10.640) ** |
| Information viewed by others | 19.035 (7.347~25.319) ** | 8.834 (4.617~16.905) ** |
| Privacy setting |  |  |
| Weibo | 3.333 (1.168~9.510) * | 0.906 (0.523~1.569) |
| QQ | 0.920 (0.447~1.895) | 0.815 (0.504~0.916) * |
| Tik Tok | 0.511 (0.179~0.757) * | 0.520 (0.190~0.688) * |
| Social media platform |  |  |
| QQ | 0.538 (0.150~1.936) | 0.569 (0.280~0.760) ** |
| Tik Tok | 0.727 (0.355~1.492) | 0.735 (0.463~0.868) * |
| Purposes of using social media |  |  |
| Because a lot of my friends are on them | 3.192 (1.446~7.046) * | 1.092 (0.622~1.916) |
| Category of information online you prefer |  |  |
| Services | 2.224 (1.024~4.829) * | 0.666 (0.402~1.105) |
| News | 0.550 (0.270~1.124) | 0.640 (0.392~1.945) |
| Products | 1.296 (0.553~3.040) | 0.355 (0.169~0.743) * |
| Purposes of updating social feed |  |  |
| Follow others | 4.076 (1.525~10.898) ** | 2.796 (1.412~5.535) * |
| People who interact most frequently on social media |  |  |
| Parents | 0.295 (0.113~0.771) * | 1.391 (0.832~2.326) |
| The motivations for interacting with others most frequently | | |
| Share information | 1.058 (0.196~1.167) | 1.668 (1.349~1.936) * |
| Time spent on social media (h) |  |  |
| 0~2 | Ref | Ref |
| 2~4 | 1.210 (0.540~2.711) | 1.096 (0.542~2.218) |
| 4~6 | 1.432 (0.578~3.549) | 1.110 (0.530~2.323) |
| 6~8 | 2.186 (0.566~8.445) | 1.811 (1.279~4.627) * |
| 8~ | 1.458 (0.353~6.015) | 2.318 (1.213~5.303) * |
| Hosmer and Lemeshow Test | 0.615 | 0.325 |

*: *P*<0.05; **: *P*<0.001
