# Supplementary material for: Exploring Association Between Social Media Addiction, Fear of Missing Out, and Self-Presentation Online Among University Students: A Cross-Sectional Study
Source: Front Psychiatry. 2022 May 13;13:896762. doi: 10.3389/fpsyt.2022.896762 (PMC9136033; doi:10.3389/fpsyt.2022.896762)
Supplement: Supplementary file 4 [file Table_4.docx]

Table S4 Comparison of variables by gender across SMA

|  | Male (n = 845) | Female (n = 1899) |
| --- | --- | --- |
|  | OR (95%CI) | OR (95%CI) |
| FoMO | 5.872 (3.964~8.698) ** | 3.780 (2.983~4.791) ** |
| Expression willingness | 1.945 (1.289~2.934) * | 1.663 (1.309~2.114) ** |
| Basic information shown on social media | 1.740 (0.935~3.238) | 0.939 (0.607~1.454) |
| Information viewed by others | 1.026 (0.699~1.507) | 1.582 (1.268~1.973) ** |
| Purposes of using social media |  |  |
| Playing games | 1.442 (1.184~2.113) ** | 1.196 (0.950~1.507) |
| Shopping | 0.994 (0.671~1.472) | 0.738 (0.571~0.955) * |
| Number of social media accounts |  |  |
| 0~2 | Ref | Ref |
| 3~4 | 0.861 (0.583~1.271) | 1.239 (0.966~1.589) |
| 5~6 | 1.681 (0.933~3.028) | 1.211 (0.873~1.682) |
| 7~8 | 0.595 (0.247~1.433) | 1.082 (0.623~1.879) |
| 9~ | 1.074 (0.492~2.345) | 1.762 (1.035~2.997) * |
| Browsing social media before going to bed | | |
| Strong disagree | Ref | Ref |
| Disagree | 1.234 (0.506~3.008) | 1.422 (0.440~4.592) |
| Not agree | 1.516 (0.659~3.488) | 1.990 (0.643~6.157) |
| Agree | 1.318 (0.588~3.116) | 2.604 (1.836~8.114) * |
| Strong agree | 1.288 (0.537~3.089) | 3.558 (1.133~11.175) * |
| Time spent on social media (h) |  |  |
| 0~2 | Ref | Ref |
| 2~4 | 1.165 (1.113~2.646) * | 1.252 (1.092~1.757) * |
| 4~6 | 1.790 (1.087~2.958) * | 1.369 (1.031~2.256) * |
| 6~8 | 2.228 (1.471~3.374) ** | 1.400 (1.077~2.005) * |
| 8~ | 2.292 (1.179~5.363) ** | 1.656 (1.064~2.577) * |
| Do you spend more time on social networking than real world? | | |
| Less | Ref |  |
| The same | 1.304 (0.982~2.439) | 1.505 (1.124~2.015) * |
| Slightly | 1.548 (1.081~2.123) * | 1.979 (1.447~2.707) ** |
| Much | 2.358 (1.235~4.196) * | 2.093 (1.413~3.101) ** |
| Hosmer and Lemeshow Test | 0.527 | 0.280 |

*: *P*<0.05; **: *P*<0.001
